# Spatial modeling of homicide mortality in the Northeast region of Brazil

**DOI:** 10.1590/0034-7167-2022-0182

**Published:** 2023-02-06

**Authors:** Carlos Carvalho da Silva, Katyucia Oliveira Crispim de Souza, Wandklebson Silva da Paz, Antônio Pedro Santana Santos, Luís Ricardo Santos de Melo, Álvaro Francisco Lopes de Sousa, Damião da Conceição Araújo, Allan Dantas dos Santos

**Affiliations:** I Universidade Federal de Sergipe. Aracaju, Sergipe, Brazil; II Universidade de São Paulo. São Paulo, Brazil; III Universidade Federal de Pernambuco. Recife, Pernambuco, Brazil; IV Universidade de São Paulo. Ribeirão Preto, São Paulo, Brazil

**Keywords:** Homicide, Social Determinants of Health, Epidemiology, Spatial Analysis, Ecological Studies, Homicidio, Determinantes Sociales de la Salud, Epidemiología, Análisis Espacial, Estudios ecológicos, Homicídio, Determinantes Sociais da Saúde, Epidemiologia, Análise Espacial, Estudos Ecológicos

## Abstract

**Objective:**

To analyze the spatiotemporal distribution of homicide mortality and association with social determinants of health in the Northeast Region of Brazil.

**Methods:**

Ecological study with spatiotemporal modeling of homicide deaths between 2000 and 2019. Temporal trends were analyzed by segmented linear regression. Crude mortality was calculated and adjusted by smoothing the local empirical Bayesian method and analyzed by the Global/Local Moran Index and spatiotemporal scan statistics. The association between social determinants of health and homicide mortality was performed using multiple linear regression and autoregressive spatial models.

**Results:**

353,089 deaths were recorded. Mortality increased from 2000 to 2019, with an annual increase of 4.37 in males and 3.57 in females. High risk spatial and spatiotemporal clusters were identified in the coastal region of the states. The spatial regression model showed an association with socioeconomic inequalities.

**Conclusions:**

High risk areas for homicides associated with socioeconomic inequality, which should be considered as a priority for designing and investing in public health policies were investigated.

## RESUMO

**Objetivo:**

Analisar a distribuição espaçotemporal da mortalidade por homicídios e associação com determinantes sociais da saúde na Região Nordeste do Brasil.

**Métodos:**

Estudo ecológico com modelagem espaço temporal dos óbitos por homicídios entre 2000 e 2019. As tendências temporais foram analisadas por regressão linear segmentada. A mortalidade bruta foi calculada e ajustada pela suavização do método bayesiano empírico local e analisadas pelo Índice de Moran Global/Local e estatística de varredura espaço temporal. A associação entre os determinantes sociais da saúde e a mortalidade por homicídios foi realizada por meio da regressão linear múltipla e modelos espaciais autorregressivos.

**Resultados:**

Foram registrados 353.089 óbitos. A mortalidade aumentou de 2000 para 2019, com um acréscimo anual de 4,37 no sexo masculino e 3,57 no feminino. Aglomerações espaciais e espaçotemporais de alto risco foram identificadas na região costeira dos estados. O modelo de regressão espacial demonstrou associação com as desigualdades socioeconômicas.

**Conclusões:**

Identificamos áreas de alto risco para homicídios associadas com a desigualdade socioeconômica, devendo ser consideradas como prioridade para o delineamento e investimento em políticas públicas em saúde.

## RESUMEN

**Objetivo:**

Analizar distribución espaciotemporal de mortalidad por homicidios y relación con determinantes sociales de salud en el Noreste brasileño.

**Métodos:**

Estudio ecológico con modelado espaciotemporal de óbitos por homicidios entre 2000 y 2019. Las tendencias temporales fueron analizadas por regresión lineal segmentada. La mortalidad brutal fue calculada y acordada por moderación del método bayesiano empírico local y analizadas por Índice de Moran Global/Local y estadística de barrido espaciotemporal. La relación entre determinantes sociales de salud y mortalidad por homicidios fue realizada mediante la regresión lineal múltiple y modelos espaciales autorregresivos.

**Resultados:**

Fueron registrados 353.089 óbitos. La mortalidad aumentó de 2000 para 2019, con un incremento anual de 4,37 en el sexo masculino y 3,57 y en el femenino. Aglomeraciones espaciales y espaciotemporales de alto riesgo fueron identificadas en la región costera de los estados. El modelo de regresión espacial demostró relación con las desigualdades socioeconómicas.

**Conclusiones:**

Identificamos áreas de alto riesgo para homicídios relacionadas con la desigualdad socioeconómica, debiendo ser consideradas como prioridad para el delineamiento e inversión en políticas públicas en salud.

## INTRODUCTION

Violence is a serious problem and constitutes a challenge for the health sector due to the impact and negative repercussions in all areas of life, both individual and collective. It is a multifactorial phenomenon related to historical, cultural, contextual, structural and interpersonal factors^(1)^. Violent actions are not only related to physical force, but also to attitudes that compromise the victim’s emotional and psychological or even any otherwise that may cause suffering to the individual and his/her family^(2)^.

Homicide is characterized as the maximum manifestation of interpersonal violence, being the main preventable cause of death, which opposes the concept of irrationality^(3)^. This phenomenon generates potential losses within the scope of public policies and highlights structural deficiencies in economic and social development, imposing the need for effective strategies to prevent and reduce the number of deaths. An average of 408,583 people worldwide were victimized by homicides in 2015, which represents a homicide mortality rate (HMR) of 5.5 per 100,000 inhabitants, and an estimated 34% occurred in the Americas region^(4)^.

Considering that homicide is a consequence of interpersonal violence, it is observed that more people die as a result of this problem worldwide than in all wars combined since 2000. At a global level, the homicide mortality rate is 6.7 per 100 thousand inhabitants per year^(5)^. Latin America is the most violent region in the world, with a homicide mortality rate six times that of Europe. In 2016, of the ten most violent countries in the world, eight are in Latin America. Brazil is one of the countries that reach the mark of tens of thousands of homicides per year^(6)^.

These high data occur due to existing problems in society, such as inequality, organized crime, impunity, corruption and private spaces of coexistence that favor domestic violence^(3)^. The Atlas of Violence 2017, produced by the Institute for Applied Economic Research (IPEA), shows that, in 2015, there were 59,080 homicides in Brazil, which corresponds to a rate of 28.9 per 100,000 inhabitants^(7)^.

Among the regions of Brazil, the North and Northeast had an increase in homicide mortality rates in the last decade, while the South, Southeast and Midwest regions had a decrease. The Northeast region suffers from the exacerbation of violence, with notoriety for homicides^(8)^. Crime in the last decade is not only a consequence of poverty, but also of scruffy and rapid development. The state with the highest homicide rate in the Northeast, in 2017, was Rio Grande do Norte (67.4), followed by Ceará (64), Pernambuco (62.3), Sergipe (58.9), Bahia (55,3), Alagoas (53.9), Paraíba (33.9), Maranhão (31.9) and Piauí (20.9)^(7)^.

Several factors are associated with homicides, such as psychological or biological dysfunctions at the individual level, or criminogenic factors such as weapons and psychotropic drugs, which can influence interpersonal conflicts and the use of lethal violence. Therefore, elements of a social, demographic and economic order — for example, income, socioeconomic inequality, population concentration and age structure — are determinants of the actions of individuals^(9)^. In addition to the factors already mentioned, it is possible to say that the occurrence of the disease may be linked to fragile family relationships and poor school performance^(10)^. Socio-economic, political, and ethnic-racial vulnerability, whether or not associated with other factors, is the basis of sociological conceptions of crime. Therefore, criminality should not be analyzed only considering the probable perpetrator of the criminal act, but also under the perspective of the reasons and processes^(3)^.

In this context, interpersonal violence is one of the main reasons for death from external causes in Brazil, becoming a challenge for national public health issues, because it impacts all areas of the lives of those involved, groups and nations. Furthermore, there are variations in the spatial distribution of mortality from interpersonal violence, as well as differences by gender, age group and ethnic and social class influences; and few studies have sought to assess and identify the causal dynamics and spatial patterns of this phenomenon in the Northeast Region, where it has shown an expressive growth. Having said that, it is understood that the risk stratification of the occurrence of deaths from aggression and the knowledge of the space and the tendency with the environment lead to the accurate analysis of the dynamics of the disease.

## OBJECTIVE

To analyze the spatiotemporal distribution of homicide mortality and association with social determinants of health in the Northeast Region of Brazil.

## METHODS

### Ethical aspects

As this is a study with data in the public domain, there was no need to submit the project to the Research Ethics Committee.

### Study design, period, and location

This is an ecological study of deaths from homicides reported in the period from 2000 to 2019, whose units of analysis were the 1,794 municipalities in the Northeast Region of Brazil. It is politically and administratively divided into nine Federative Units (Alagoas, Bahia, Ceará, Maranhão, Paraíba, Pernambuco, Piauí, Rio Grande do Norte and Sergipe), representing 18% of the country’s territory. Its territorial extension covers an area of 1,554,257 km2 and has a population of 53,081,654 inhabitants^(11)^. It is important to emphasize that it is still the scene of important socioeconomic disparities, portrayed through the lowest human development index (HDI) in the country (0.608); and almost half of its municipalities (47.7%) have a high social vulnerability index (SVI), while 32.4% are in the range of very high social vulnerability^(12)^.

### Population, inclusion, and exclusion criteria

The study population comprised all deaths from homicide reported in the Mortality Information System (MIS) of the Department of Informatics of the Unified Health System (DATASUS) of the Health Surveillance Secretariat of the Ministry of Health (SVS/MS), in the period from 2000 to 2019, of residents in the municipalities of the Northeast Region.

Death by homicide was considered as any record with the final classification of homicides resulting from aggressions caused by third parties, using various means that can cause damage, injury, or death of the victim, according to the Tenth Revision of the International Statistical Classification of Diseases and Health Related Problems (ICD-10).

We included records of deaths classified as aggression (ICD-10: X85-Y09) in Chapter XX of the Tenth Revision of the International Statistical Classification of Diseases and Related Health Problems (ICD 10). Deaths without identification of the municipality of residence and duplicates with two or more records for the same case were excluded.

### Variables and data source

The dependent variable of this study was homicide mortality in the municipalities of the Northeast Region, reported and collected in the Mortality Information System (MIS) of the Department of Informatics of the Unified Health System (DATASUS) of the Health Surveillance Secretariat of the Ministry of Health (SVS/MS). The covariates were the social determinants of health collected from the 2010 demographic census of the Brazilian Institute of Geography and Statistics (IBGE) and the United Nations Program (UNDP).

- Sociodemographic variables: monthly average nominal family income; Gini index; unemployment rate; municipal human development index (MHDI); MHDI income; MHDI Longevity; MHDI Education; illiteracy rate - 18 years of age or older; proportion of the population living in households with a density greater than two people per bedroom; proportion of poor; proportion of vulnerable to poverty;
- Vulnerability variables: dependency ratio; proportion of people between 15 and 24 years of age who do not study, do not work and are vulnerable, in the vulnerable population of this age group; proportion of mothers who are heads of households, without complete elementary education and with at least one child under 15 years of age, in the total number of mothers who are heads of households and with a minor child; proportion of people in households that are vulnerable to poverty and who spend more than one hour commuting to work.

### Study protocol

The study followed the guidelines of the Strengthening the Reporting of Observational Studies in Epidemiology initiative (STROBE)^(13)^.

### Analysis of results and statistics

The gross coefficients of mortality from homicides according to sex and Federative Unit were calculated through the number of deaths from homicides (deaths from aggression - ICD10 Group: X85-Y09), per 100,000 inhabitants, in the population residing in a given geographic space, in the considered year.

Temporal trends were analyzed using joinpoint regression models (segmented linear regression). Mortality coefficients were considered dependent variables; and the year of the event, as an independent variable. This method allows verifying changes in the indicator trend over time by adjusting data from a series based on the smallest number of possible joinpoints (the value 0 indicates a straight line without inflection points) and tests whether the inclusion of more joinpoints is statistically significant. Thus, time series can present an increasing, decreasing or stationary trend and even different trends in sequential sections^(14)^.

The gross homicide mortality coefficients were smoothed by the local empirical Bayesian smoothing method to minimize the instability caused by the random fluctuation of rates, especially in municipalities with small populations and few events^(15)^. The Monte Carlo permutation test was used to choose the best segment for each model. The model that presented the highest coefficient of residue determination (R2) was considered the best model. Then, the annual percentage change (APC – annual percent change) and its respective confidence interval (95%CI) were calculated for each segment in order to describe and quantify the trend, in addition to assessing whether it is statistically significant. The average annual percentage chance (AAPC) for the entire period was calculated with the aim of simplifying the comparison of trends for indicators with more than one significant slope in the period. Its estimate is obtained by the weighted geometric mean of the APC, with the weights equal to the length of each time interval of the segment. The trends will be statistically significant when APC and AAPC present p value < 0.05^(16)^.

Subsequently, the analysis of spatial autocorrelation by the Moran Global Index was used in order to investigate the existence of patterns of occurrence of the phenomenon studied in space. A spatial proximity matrix was created using the contiguity criterion, and the Global Moran Index was calculated. This index estimates the correlation of a variable with itself in space, ranging from -1 to +1, where values close to 0 indicate spatial randomness; positive values, positive spatial autocorrelation; and negative values, negative autocorrelation. The results with p < 0.05 demonstrate regions where there are spatial structures for the occurrence of deaths by homicide^(16)^.

Subsequently, the occurrence of local spatial autocorrelation (Local Indicators of Spatial Association - LISA) was evaluated using the Local Moran Index, which determines the dependence of local data on its neighbors and enables the identification of spatial association patterns which may indicate the occurrence of spatial clusters of municipalities^(17)^. The Moran scattering diagram, based on the Local Moran Index, was used to identify critical or risk areas and transition areas, aiming to compare the value of each municipality with its neighbors and verify the existence of spatial dependence, in addition to identifying patterns spatial.

This diagram was represented using the Moran Map, in which only municipalities with statistically significant differences (p < 0.05) were considered. Thus, the spatial quadrants were generated: a) Q1 (high/high or hotspots – positive values, positive averages) and Q2 (low/low or coldspots – negative values, negative averages) indicate points of positive spatial association or similar to their neighbors, that is, areas of agreement for homicide; b) Q3 (high/low – positive values, negative means) and Q4 (low/high – negative values, positive means) indicate points of negative spatial association, that is, transition areas^(15)^.

The scanning statistic (SatScan) was applied to identify spatiotemporal clusters (existence of simultaneous spatial and temporal proximity between deaths) at high risk for the occurrence of homicide, whose numbers were recorded by municipality of residence and population estimate for the study period. served as a basis. The KullDorf method of retrospective analysis was used, and the Poisson distribution model was applied to detect and evaluate spatiotemporal clusters of homicide deaths. The following parameters were considered: aggregation time of one-year, non-overlapping clusters, circular clusters, maximum spatial cluster size of 50% of the population at risk and maximum temporal cluster size of 50% of the study period. The primary and secondary clusters were detected using the likelihood ratio test. Results were considered significant when p < 0.05 using 999 Monte Carlo simulations^(17)^.

The association between social determinants of health and homicide mortality was investigated using multiple linear regression and spatial autoregressive models. Initially, we calculated the natural logarithm (Ln) of the smoothed Bayesian homicide mortality rate as the dependent variable, in order to make the distribution of the dataset more consistent with a normal distribution. For the construction of the model, the Spearman correlation between the dependent variable and the independent variables was applied, aiming to select the positive and negative correlations with a significance of p > 0.20.

Then, multiple linear regression analysis was performed in order to select the variables that could be the most explanatory factors. All pre-selected variables were incorporated into the model and were excluded at a significance level of p < 0.05 (stepwise selection). The model obtained was tested using the Lagrange multiplier statistical test for diagnosis of spatial dependence and decision on the spatial regression model to be used: spatial error and spatial lag models. The model’s performance was evaluated using the likelihood function^(18)^. The model with the best fit was determined using the Akaike information criterion (AIC) and the Bayesian information criterion (BIC). The residuals of the models were analyzed using Moran Global I and Moran Local I in order to test whether the spatial autocorrelation was eliminated after the application of the models^(19)^.

In the elaboration of the maps, the cartographic base of the Northeast Region from the IBGE was used, which is available in digital media. The cartographic projection used corresponded to the Universal Transverse Mercator (UTM) system, Terra Datum horizontal model SIRGAS 2000 and the 24S spindle.

The following software were used for data analysis and processing: Microsoft Office Excel 2016, Microsoft Corporation, Redmond, WA (USA), Jamovi 2.2.5, TerraView 4.2.2 (Instituto Nacional de Pesquisas Espaciais, INPE, SP, BR), Joint Point Regression 4.3.1.0 (US National Cancer Institute, Bethesda, MD, USA), QGIS (Open Source Geospatial Foundation, OSGeo, CHI, US, Version 2.18.2), GeoDa, version 1.14.0 (Spatial Analysis Laboratory, University of Illinois at Urbana-Champaign, USA) and SaTScan 9.1.1 (Harvard Medical School, Boston and Information Management Service Inc., Silver Spring, MD, USA).

## RESULTS

In the period from 2000 to 2019, a total of 353,089 deaths from homicides were recorded in the Northeast of Brazil. The predominant characteristics of the cases in the region were: male (93.04%), between 20 and 29 years of age (40.03%), of mixed race (75.8%), with low or no schooling (53%), single (71.4%) and that took place on public roads (50.7%).

The temporal trend of mortality for the Northeast showed an increase for males with an annual increase of 4.37 (95%CI: 3.32 to 5.51) and for females with an annual increase of 3.57 (95% CI: 2.62 to 4.62). An increasing trend was observed in all states with the exception of Pernambuco (Table 1).

**Table 1:** Temporal trends in homicide mortality by gender in the Northeast Region of Brazil, from 2000 to 2019, Northeast, Brazil.

Variables	Period	APC	CI 95%	Trend	p
Men					
North East	2000-2019	4.37*	3.32; 5.51	Increasing	< 0.001
Maranhão	2000-2019	7.07*	5.01; 9.17	Increasing	< 0.001
Piauí	2000-2019	5.02*	3.79; 6.25	Increasing	< 0.001
Ceará	2000-2019	7.07*	4.81; 9.39	Increasing	< 0.001
Rio Grande do Norte	2000-2019	10.61*	8.46; 12.8	Increasing	< 0.001
Paraíba	2000-2019	4.58*	2.27; 6.95	Increasing	0.001
Pernambuco	2000-2019	-1.36*	-2.51; -0.2	Decreasing	0.025
Alagoas	2000-2019	2.50*	0.06; 4.99	Increasing	0.025
Sergipe	2000-2019	5.73*	4.13; 7.36	Increasing	< 0.001
Bahia	2000-2019	6.60*	4.61; 8.63	Increasing	< 0.001
Women					
North East	2000-2019	3.57*	2.62; 4.62	Increasing	< 0.001
Maranhão	2000-2019	5.57*	3.59; 7.59	Increasing	< 0.001
Piauí	2000-2019	3.73*	2.03; 5.46	Increasing	< 0.001
Ceará	2000-2019	6.78*	4.67; 8.93	Increasing	< 0.001
Rio Grande do Norte	2000-2019	8.27*	6.4; 10.17	Increasing	< 0.001
Paraíba	2000-2019	3.85*	0.98; 6.83	Increasing	0.011
Pernambuco	2000-2019	-1.88*	-2.69; -1.06	Decreasing	< 0.001
Alagoas	2000-2019	1.68*	-0.57; 3.98	Increasing	0.035
Sergipe	2000-2019	3.16*	1.23; 5.13	Increasing	0.003
Bahia	2000-2019	5.68*	3.74; 7.66	Increasing	< 0.001

*APC: annual percentage change; 95% CI: 95% confidence interval*

The spatial distribution of homicide mortality in the male population was more concentrated in the coastal regions of the states of Bahia, Sergipe, Alagoas, Pernambuco and Ceará. The analysis of spatial autocorrelation revealed clusters with high-high pattern in 321 municipalities, belonging to the states of Bahia, Sergipe, Alagoas, Pernambuco, Paraíba, Rio Grande do Norte and Ceará; and with a low-low standard in 461 municipalities, mainly in Maranhão, Piauí and central and northern Bahia.

Regarding the spatial distribution of homicides in the female population, a higher concentration was also observed in the states of Pernambuco, Alagoas, Sergipe, Bahia and Ceará. Smoothed rates showed values from 3% to 6% per 100,000 inhabitants in all states. High-risk clusters were detected in 214 municipalities in the states of Bahia, Sergipe, Alagoas, Pernambuco, Paraíba, Rio Grande do Norte and Ceará.

**Figure 1:**
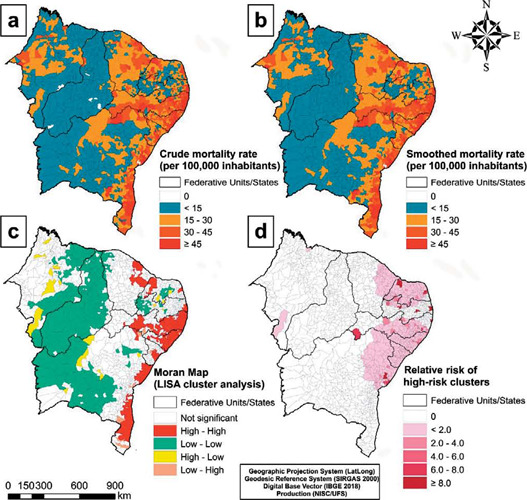
Spatial and spatiotemporal distribution of homicide mortality in the general population in the Northeast Region of Brazil in the period 2000-2019

In the spatiotemporal analysis, eight statistically significant spatiotemporal clusters of homicide mortality were identified (Table 2 and Figure 1D). The primary cluster included the highest number of deaths (14,538) between the years 2000 and 2009 of the municipalities in the states of Pernambuco, with a gross annual rate of 2,353.61/100 thousand (RR = 74.81; p < 0.001). It is noteworthy that municipalities in Paraíba were part of four of the eight identified clusters; and this state was responsible for the second cluster (2007-2016) with the highest annual mortality (6,010/100 thousand) and the second highest relative risk (RR = 53.47; p < 0.001) (Table 2 and Figure 1). The observed clustering pattern corroborates the findings of the univariate LISA analysis and the smoothed rate distribution pattern by the local empirical Bayesian method (Figure 1D).

**Table 2:** Spatiotemporal clusters of annual homicide mortality rates in the general population between 2000 and 2019

Clusters	Period	States	District	Deaths	Expected deaths	Annual mortality rate*	RR	LLR	P
1	2000-2009	PE	3	14,538	202.67	2,353.61	74.81	48,083.49	< 0.001
2	2007-2016	PB	3	6,010	114.33	1,724.86	53.47	17,966.83	< 0.001
3	2009-2018	PB, PE, AL, SE and BA	391	74,375	49,245.97	49.62	1.65	6,620.26	< 0.001
4	2012-2018	CE, RN and PB	246	34,347	17,709.94	63.64	2.04	6,539.28	< 0.001
5	2007-2016	PB	1	1,638	18.75	2,866.21	87.76	5,706.32	< 0.001
6	2000-2009	PE	1	1,232	38.59	1,047.67	32.04	3,075.66	< 0.001
7	2012-2016	MA	4	4,585	2,264.13	66.42	2.04	922.09	< 0.001
8	2017-2019	MA	1	140	92.9	49.43	1.51	10.31	0.952

MA – Maranhao; EC – Ceara; RN – Rio Grande do Norte; PB – Paraíba; PE – Pernambuco; AL – Alagoas; SE – Sergipe; BA – Bahia; RR – relative risk for the cluster compared to the rest of the region; LLR – likelihood ratio. * Homicide mortality coefficient (per 100,000 inhabitants) during the grouping period.

*(A) Crude homicide mortality rate; (B) Smoothed homicide mortality rate; (C) Univariate LISA analysis; (D) Spatiotemporal scan analysis.*

The spatial lag model presented the best fit when compared to the multiple linear regression model, obtaining the highest values for the likelihood function and explanatory power (R2 = 0.83; p = 0.01) and the lowest values for ACI and BCI The factors associated with homicide mortality in the general population were: Gini index, unemployment rate, income, education, proportion of the poor and proportion of people in households vulnerable to poverty and who spend more than one hour commuting (Table 3).

**Table 3:** Results and comparison of multiple linear regression models and spatial lag of the Bayesian smoothed rate logarithm of homicide mortality in the general population and social determinants of health

Social determinants of health	Multiple linear regression model		Spatial lag model	
	Coefficient	p	Coefficient	p
Average monthly nominal family income	0.49	0.81	0.80	0.94
Gini index	-0.51	0.01	-0.74	< 0.01
Unemployment rate	-3.91	0.00	-0.61	0.04
Municipal Human Development Index - MHDI	0.87	0.00	2.69	0.09
MHDI Income	0.37	0.03	0.35	0.00
MHDI Longevity	0.11	0.00	-0.32	0.81
MHDI Education	0.19	0.71	-0.76	< 0.01
Illiteracy rate - 18 years of age or older	0.35	0.59	0.26	0.09
Proportion of the population living in households with a density greater than two persons per bedroom	-0.48	0.00	-0.63	0.15
Proportion of poor	0.10	0.00	0.34	0.01
Proportion of vulnerable to poverty	-0.12	0.10	-0.45	0.09
Dependency reason	-0.20	0.07	0.26	0.66
Proportion of people aged 15 to 24 who do not study, do not work and are vulnerable, in the vulnerable population of this age group	-0.20	0.10	2.3	0.97
Proportion of mothers who are heads of households, without complete elementary education and with at least one child under 15 years of age, in the total of mothers who are heads of households and with a child underage	0.27	0.10	0.19	0.08
Proportion of people in households that are vulnerable to poverty and spend more than one hour commuting to work	0.27	0.05	0.46	0.01

Model evaluation criteria		
Akaike Information Criterion	2.880.94	1.027.98
Bayesian Information Criterion	2.968.72	1.121.24
Log-likelihood	-1.424.47	-1.136.66
Determination coefficient (R^2^)	0.40 (p=0.01)	0.83 (p=0.01)
Global Moran Index of Regression Residual	0.60 (p=0.01)	-0.45 (p=0.67)

## DISCUSSION

This study showed an integrated analysis of homicide mortality in Northeast Brazil, over a period of 20 years, and its association with the social determinants of health, using temporal, spatial, spatiotemporal analysis techniques, and classical and spatial regression models.

The results revealed the patterns of distribution of homicide mortality in the municipalities of the Northeast Region. These data can be useful for planning public health policies to combat violence. Other similar studies, which used geotechnologies to understand the spatial dynamics related to the occurrence of the disease, were also carried out in Brazil^(20)^. An increase in mortality was identified in the period 2000-2019, demonstrating that homicides are a worrying public health problem.

This increase may be related to the urbanization process and inadequate allocation of financial resources directed to the development of public health measures to reduce the disease^(21)^. Other factors that may favor homicide mortality in the general population in the Northeast are the accelerated and exponential growth of the population, driven by the rural exodus, which increased the number of people on the periphery of the Northeastern capitals; low education and low income, which trigger social inequality in these areas; as well as the cultural and anthropological issue as strong drivers of the extermination of young people^(22)^.

The temporal trend analysis showed that the increase in mortality occurred in both sexes. As for sociodemographic characteristics, a higher occurrence was observed in males, aged between 20 and 29 years old, of mixed race and low or no schooling, corroborating a study carried out in Rio Grande do Norte, which observed an increase in the number of deaths and the same epidemiological profile^(22)^.

The high mortality rate from homicides in males may be related to the adoption of lifestyles linked to violence, such as the use of alcohol, illicit drugs, participation in organized crime and the use of firearms, especially in the group of young adults. Associated with this, social and cultural spaces contribute to the construction of the concept of masculinity related to violence and the perpetuation of patriarchal culture^(3, 23, 24)^.

In the female population, although not very accentuated, it is important to point out that this increase can be attributed to the increase in cases of femicide in the Northeast Region. Studies indicate that the profile of women victims of femicide is young, of childbearing age, with low education^(25)^. Thus, the high social hierarchy makes it difficult for women to perform social functions that guarantee them freedom and autonomy, which is a potential catalyst for homicides that lead to femicide^(26)^. Despite the fact that femicide was declared a crime in 2015 and the decision of the Maria da Penha Law (MPL) that established support services for women and more severe punishment for aggressors, the crime persists, generating numerous problems in different social and cultural contexts. The factor that hinders the effectiveness of MPL in all spaces is the concentration of services in state capitals, as well as the absence of tools and policies that bring information to the entire population^(27)^.

A study on the mortality of Brazilian adolescents and young adults between the years 1990 and 2019 shows an increase in mortality rates in the North and Northeast and a reduction in the Southeast and South states, where[?] At the national level, the first cause [occurred in what state?] of death in females were transport injuries, followed by interpersonal violence; and, for males, interpersonal violence was the leading cause of death, especially in the Northeast^(28)^. Premature deaths generate psychophysical and socioeconomic consequences, considering, in particular, the loss of young people in their productive phase^(29)^. It was also shown that most of these homicides occur on public roads, followed by the hospital.

The spatial and spatiotemporal analysis identified high-risk clusters for homicide deaths in municipalities located in the coastal region. Previous studies showed similar results^(30, 31)^. Due to this scenario, the states of Pernambuco and Paraíba promoted Public Security programs aimed at reducing violence and deaths, known as the “Pact for Life” and “Paraíba United for Peace”, respectively^(31, 32)^.

The state of Pernambuco was the only one in the Northeast region that showed a decreasing trend for both sexes in the last two decades. This reduction may be the result of the implementation of the Pact for Life (PPV), a policy instituted in 2007, which, by 2013, had reduced the number of homicides in the state by 40%^(32)^. It is important to highlight that, despite being considered a successful policy, the reduction in mortality observed by the Pact for Life was lower or non-existent in female victims^(33)^. Also, it is worth mentioning that both Pernambuco and Paraíba had the highest mortality rates in the region.

The model with the best performance to explain the association between homicide mortality and the social determinants of health was the spatial lag model. The spatial model reveals an adequate structure to study the relationships of factors that contribute to the occurrence of health problems. Thus, determinants such as higher income inequality (Gini index), high unemployment rate, low income, low education, higher proportion of poor people and people in households that are vulnerable to poverty and who spend more than an hour to get to work showed association with homicide mortality. Considering that the number of deaths from aggression is higher in low-income areas and higher income inequality, it is necessary that health policies target specific multisectoral actions, resulting in a reduction in the number of homicides in these communities^(34)^.

The importance of social determinants of health (SDH) for people’s health situations was observed over time, as the biomedical model predominated. It was only at the Alma-Ata conference, in the 1970s, that questions about vertical health models occurred, highlighting the SDHs as factors that influence the emergence of diseases^(35)^.

With the exception of Teresina, all the capitals of the Northeast are located in coastal regions recognized as major cosmopolitan and tourist centers. Inequality in the structuring of cities, when not associated with the promotion of public policies that meet the needs of the population, are capable of generating inequalities conducive to the emergence of pockets of poverty, spaces built without planning and investment in public policies, favoring the increase in violence, marginalization and dispute over territories^(36)^.

It is observed that socioeconomic inequalities in the municipalities also make it difficult both to access adequate information regarding the legislation and to understand that violence is a public health problem due to its magnitude, deaths, and costly expenses for the public system^(25, 37)^. Thus, as it is a multifactorial problem, policies aimed at reducing homicide mortality must be comprehensive, focusing on structural determinants and based on the characteristics of each territory, such as unemployment, income inequality and poverty concentration, advocating preventive actions and income distribution, to the detriment of those purely ostensible^(38)^.

### Study limitations

There is a possibility that the data are underestimated due to incompleteness in filling out death certificates or underreporting of deaths from homicides in the Mortality Information System.

### Contributions to the area of Health or Public Policy

This study highlights the contribution of an integrated spatiotemporal analysis to identify high-risk areas and associated factors for homicide mortality. The results can improve the management of health services to develop strategies and public policies that reduce violence and homicides in the general population. The analysis of the social determinants of health related to homicides made it possible to point out the need for a broader discussion for the construction of intersectoral actions aimed at reducing inequalities and, consequently, inequities in health.

## CONCLUSIONS

The data from this study allowed a broad characterization of homicide mortality in the Northeast Region. It is observed that this is a persistent problem and that it has a strong association with situations of social vulnerability.

High-risk areas were identified in the coastal regions of the Brazilian Northeast, with an association of socioeconomic inequality factors, and should be considered as a priority for the design and investment in public health policies.

It is suggested, therefore, the elaboration of intersectoral public policies, which integrate public security and health services and are centered on people, on interpersonal relationships and on meeting needs, for the identification of vulnerable areas and targeted action. In addition, it is expected that the data obtained and presented here will be used by other authors in order to broaden the discussion on the subject.
